# A Systematic Review and Meta-Analysis of Trifluridine/Tipiracil plus Bevacizumab for the Treatment of Metastatic Colorectal Cancer: Evidence from Real-World Series

**DOI:** 10.3390/curroncol30060397

**Published:** 2023-05-24

**Authors:** Ioannis A. Voutsadakis

**Affiliations:** 1 Algoma District Cancer Program, Sault Area Hospital, Sault Ste Marie, ON P6B 0A8, Canada; ivoutsadakis@yahoo.com; 2 Division of Clinical Sciences, Section of Internal Medicine, Northern Ontario School of Medicine, Sudbury, ON P3E 2C6, Canada

**Keywords:** TAS-102, metastatic, later line, colorectal cancer, anti-angiogenic

## Abstract

Background: Colorectal cancer is the most prevalent gastrointestinal neoplasm. When metastatic, the disease has limited systemic treatment options. Novel targeted therapies have expanded these options for subsets with specific molecular alterations, such as microsatellite instability (MSI)-high cancers, but additional treatments and combinations are in urgent need to improve outcomes and improve survival of this incurable disease. The fluoropyrimidine-derivative trifluridine, in combination with tipiracil, has been introduced as a third-line treatment, and more recently, it was studied in combination with bevacizumab. This meta-analysis reports on studies with this combination in clinical practice outside clinical trials. Methods: A literature search in the Medline/PubMed and Embase databases was executed for finding series of trifluridine/tipiracil with bevacizumab in metastatic colorectal cancer. Criteria for inclusion in the meta-analysis were English or French language of the report, inclusion of twenty or more patients with metastatic colorectal cancer treated with trifluridine/tipiracil in combination with bevacizumab outside of a trial and containing information regarding response rates, progression-free survival (PFS), and overall survival (OS). Information on the demographics of the patients and on adverse effects of treatment was also collected. Results: Eight series with a total of 437 patients were eligible for the meta-analysis. The performed meta-analysis discovered a summary response rate (RR) of 2.71% (95% confidence interval (CI): 1.11–4.32%) and a disease control rate (DCR) of 59.63% (95% CI: 52.06–67.21%). Summary PFS was 4.56 months (95% CI: 3.57–5.55 months), and summary OS was 11.17 months (95% CI: 10.15–12.19 months). Common adverse effects identified mirrored the adverse-effect profile of the two components of the combination. Conclusion: The current systematic review and meta-analysis reports the efficacy of trifluridine/tipiracil with bevacizumab in advanced lines of therapy for metastatic colorectal cancer in the setting of clinical practice outside clinical trials. Discovery of predictive biomarkers of response to trifluridine/tipiracil with bevacizumab will promote the tailoring of this treatment to individual patients to maximize clinical benefit.

## 1. Introduction

Colorectal cancer is the most prevalent gastrointestinal carcinoma and one of the most frequent cancers across the globe [1]. In the United States alone, about 150,000 patients have been diagnosed with colorectal cancer in 2022, and over 50,000 patients have been projected to die from the disease [1]. Colorectal cancer is the third-leading cause of cancer mortality in both men and women, falling behind lung and prostate cancers in men and behind lung and breast cancers in women. Most colorectal cancer patients are diagnosed with localized stage I to III disease amenable to surgical resection. De novo metastatic disease is diagnosed in about 20% of all newly diagnosed colorectal cancer cases [2]. In addition, several patients diagnosed initially with stage II and stage III disease will have a metastatic relapse, despite neo-adjuvant or adjuvant chemotherapy and radiotherapy [2]. Remarkable progress in systemic and local therapies has been made in the past decades for the treatment of metastatic colorectal cancer, but the disease remains rarely curable, mostly in the specific case of oligometastatic tumor burden amenable to resection [3]. Therapeutic advances have been introduced with the elucidation of the molecular lesions characterizing colorectal carcinogenesis, which resulted in improved survival of selected patients with the introduction of targeted therapies [4,5,6,7]. Targeted therapies currently in clinical use in colorectal cancer include the anti-EGFR monoclonal antibodies cetuximab and panitumumab, alone or with chemotherapy for *KRAS* wild-type disease; combinations of anti-EGFR monoclonal antibodies with BRAF inhibitors, such as vemurafenib and encorafenib, for colorectal cancers bearing *BRAF* V600E mutations; and anti-HER2 therapies, including trastuzumab, lapatinib, and tucatinib, for HER2-altered cancers and immune checkpoint inhibitors, such as pembrolizumab and nivolumab, for cancers that display high microsatellite instability (MSI-H). Anti-angiogenic therapies are also useful in metastatic colorectal cancers without biomarkers of response [8]. However, in most patients with metastatic colorectal cancer, chemotherapy remains the backbone of therapy. Combinations of 5-fluoropyrimidine with oxaliplatin or irinotecan constitute the first two lines of therapy in the majority of patients with microsatellite-stable metastatic disease [9]. Beyond these initial lines of treatment, the only generic systemic therapies that have been approved for later-line therapy are trifluridine/tipiracil and the small-molecule kinase inhibitor regorafenib, both providing only modest clinical benefits [10,11].

Trifluridine/tipiracil, also known as TAS-102, is available as an oral combination formulated in a single tablet with the two components in a fixed 1:0.5 molar ratio [12]. Trifluridine is an anti-metabolite that can become incorporated into DNA after triphosphorylation through thymidylate synthase. Tipiracil is an inhibitor of the enzyme thymidine phosphorylase, which mediates the first-pass liver catabolism of trifluridine [12]. The combined oral administration of the two components allows for systemic therapeutic levels of trifluridine to be obtained, avoiding the first-pass liver catabolism of the anti-metabolite. Trifluridine/tipiracil in the third-line therapy of metastatic colorectal cancer was associated with an overall survival of about 7 months [13]. Objective responses were rare (1.6%, all partial responses), but about 40% of patients obtained disease stability for at least 6 weeks. In addition, the combined formulation showed acceptable toxicity and oral administration has practical advantages in the setting of later-stage advanced disease [13]. To build on the efficacy results of trifluridine/tipiracil, a combination with the anti-angiogenic agent bevacizumab, which is also successfully combined with other chemotherapy backbones in the treatment of metastatic colorectal cancer in earlier lines of therapy, has been studied in phase 2 trials and more recently in a phase 3 trial. The phase 3 randomized SUNLIGHT trial recently reported an overall survival benefit with the combination of trifluridine/tipiracil and bevacizumab over trifluridine/tipiracil alone in the third-line metastatic treatment setting [14]. The systematic review and meta-analysis reported in this article examines recently published series of the trifluridine/tipiracil and bevacizumab combination in the later-line metastatic colorectal cancer treatment with the aim to confirm whether results from this therapy outside trials are similar with the trials’ results.

## 2. Methods

A search was performed in the Medline/PubMed and Embase databases with the search terms “trifluridine tipiracil” or “lonsurf” or “TAS-102” and “bevacizumab”. Inclusion criteria for retention of retrieved articles included English or French language; describing treatment of metastatic colorectal cancer patients with the combination of trifluridine/tipiracil and bevacizumab outside a clinical trial; inclusion of at least 20 patients; and providing information or data for calculation of at least one of the four efficacy outcomes of interest: response rate (RR), disease control rate (DCR), progression-free survival (PFS), and overall survival (OS). RR was defined as the sum of complete and partial response, and DCR was defined as RR plus stable disease rate. Articles describing clinical trials, case reports, series of fewer than 20 patients, and combinations with additional drugs were excluded. Also excluded were publications with overlapping cohorts, as defined by overlapping authors, centers, and patient-recruitment dates. In these cases, the series with the greater number of patients was included and the overlapping series with the smaller number of patients was excluded. The literature search included articles published up to 28 February 2023.

A review of the reference lists of the articles automatically identified and included in the primary search was conducted manually to detect supplemental pertinent publications. The ROBINS-I tool was employed for assessing the risk of bias associated with every study included in the meta-analysis [15].

Demographics of the patient population treated in each series and the efficacy and toxicity of the trifluridine/tipiracil and bevacizumab regimen were obtained from the individual publications. The protocol-stipulated acquisition of the following demographic and molecular characteristics were outcomes of interest if available: the age of the patients; Eastern Cooperative Oncology Group (ECOG) performance status (PS); number and type of previous lines of treatment for metastatic disease; number and site of organs involved in metastatic cancer; location of the primary in the right or left colon and rectum; microsatellite instability (MSI) status; and mutation status of *KRAS*, *BRAF*, and *NRAS* genes. All grade toxicities and grades 3 and 4 toxicities were also outcomes of interest, with a focus on known toxicities of each combination component. 

Descriptive statistics, such as means, 95% confidence intervals, and standard deviations, were calculated for the various parameters of interest, the four efficacy outcome measures, and the prevalence of adverse effects. Some series included in the meta-analysis provided incomplete population data or did not present data for all outcomes. For this reason, computation of some population characteristics and outcomes of interest were based on smaller numbers of patients than the sum of the number of patients in the included series. The number of series each outcome of interest was derived from was clarified in the presentation of each outcome. Heterogeneity evaluation amongst included studies was performed with Cochran’s Q and the *I*^2^ tests. The fixed-effects model was used if between-studies heterogeneity was low. In the opposite situation, if heterogeneity was high, a random-effects model was utilized for calculation of the summary statistic [16]. Calculations for the meta-analysis were performed with the Excel tool (Microsoft Corp.), utilizing a previously published methodology with minor modifications as necessary [17].

The meta-analysis was performed according to the PRISMA guidelines, but it was not registered with PRISMA.

## 3. Results

**Figure 1:**
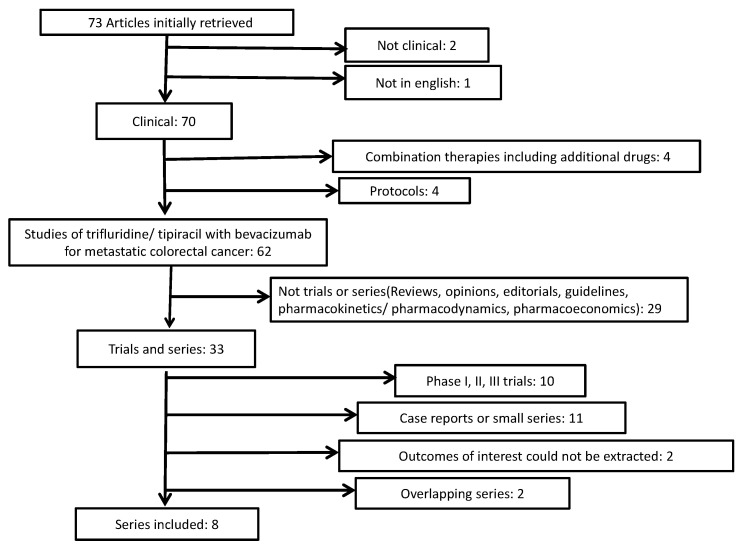
Flow diagram of the research articles screened and selected for the meta-analysis.

Seventy-three articles were obtained using the search terms, and abstracts were reviewed to determine further eligibility (Figure 1). Reasons for exclusion are depicted in the diagram of Figure 1. The main types of identified articles leading to exclusion were reviews, guidelines, and editorials. Ten series for potential inclusion in the meta-analysis were identified [18,19,20,21,22,23,24,25,26,27]. After evaluation, two of the series were excluded, as they contained, based on the authors, similar centers and dates of study performance, potentially overlapping patients [26,27]. Among the remaining eight series, six series were from Asia (of which five series were from Japan, where trifluridine/tipiracil was initially developed, and one series from China, Table 1) [18,21,22,23,24,25]. Two series were from European centers [19,20]. Dates of publication were between 2020 and 2022, and a total of 441 patients were described. The series included in the meta-analysis presented an overall moderate to high risk of potential bias, mostly related to the retrospective, non-randomized design of the studies. Domains of the ROBINS-I tool with the most significant risk for bias included selection bias; bias due to missing data, as patients with missing outcomes would have been excluded from the series; and bias due to measurement of outcomes, as no central review was performed in these retrospective studies.

Regarding baseline characteristics, the median age of patients in the included studies varied between 53 and 73 years old and slightly more patients (52.8%) were males (Table 2). Seven of the eight included series reported information on the ECOG performance status (PS) of the patients. The majority of patients (92.4%) had an ECOG PS of zero or one. Five series with 249 patients reported data on previous lines of therapy received. The range of previous lines of therapy was from one to more than four, and almost half of the patients had received more than two lines of previous therapies. All patients had received a previous fluoropyrimidine, and over 95% had received oxaliplatin, irinotecan, and at least one anti-angiogenic agent (bevacizumab, aflibercept, or regorafenib). Primary tumor sidedness was inconsistently reported in the eight series, with right colon primaries being present in about one fourth of patients and the rest of the patients having had left colon or rectal primaries (Table 2). Metastatic organ involvement was also inconsistently reported. Most frequent involvement, when this information was available, concerned the lung and the liver, followed by the peritoneum. Five series reported data on surgery for the primary tumor. About three-fourths of patients (72.5%) had previously undergone surgical resection of their primary cancer, while the rest had their primary in situ (Table 2).

Data on the microsatellite instability status of the tumors was available in three of the series with 120 patients (Table 3). Tumors were microsatellite-stable or mismatch-repair-proficient in 81.7% of cases and microsatellite-unstable or mismatch-repair-deficient in 2.5% of cases. *KRAS* status was available in all series included, and mutations were present in 51.7% of cases. *BRAF* was mutated in 3.5% of cases in the four series with 259 patients that reported this information (Table 3). *NRAS* was mutated in 7.4% of cases in the single series that included the status of this gene.

**Figure 2:**
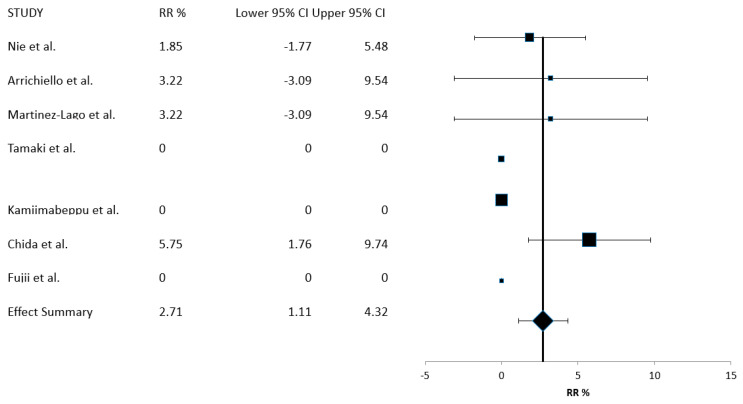
Meta-analysis of response rate (RR) [18,19,20,21,22,23,24,25].

Seven of the eight series included in the meta-analysis, with a total of 405 patients, reported data on the RR of trifluridine/tipiracil with bevacizumab treatment and were included in the calculations for this outcome. The summary RR value was 2.71% (95% CI: 1.11–4.32%) (Figure 2). All observed responses were partial, and three of the seven studies had no responding patients. Calculations for the meta-analysis of the RR were executed using a fixed-effects model, given that heterogeneity between studies was low and the *I*^2^ value was zero (Cochran’s Q = 2.03, x^2^ *p* = 0.91). 

**Figure 3:**
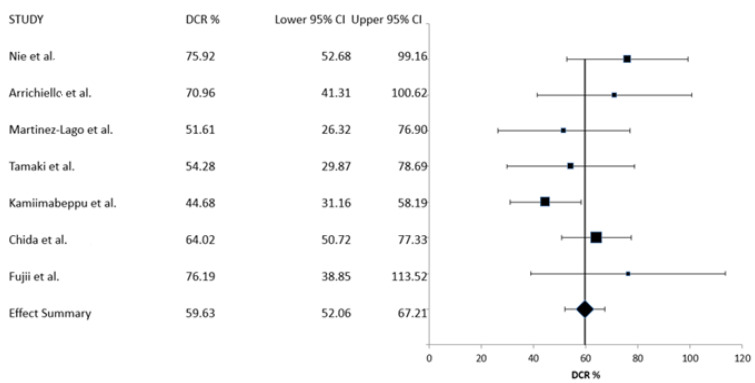
Meta-analysis of disease control rate (DCR) [18,19,20,21,22,23,25].

The same seven series that provided RR data provided also data for the DCR analysis. Summary DCR obtained with trifluridine/tipiracil with bevacizumab treatment was 59.63% (95% CI: 52.06–67.21%) (Figure 3). Between studies, heterogeneity was also low in this case (*I*^2^ = 0, Cochran’s Q = 0.0001, x^2^ *p* = 1), and thus, a fixed-effects model was retained. 

**Figure 4:**
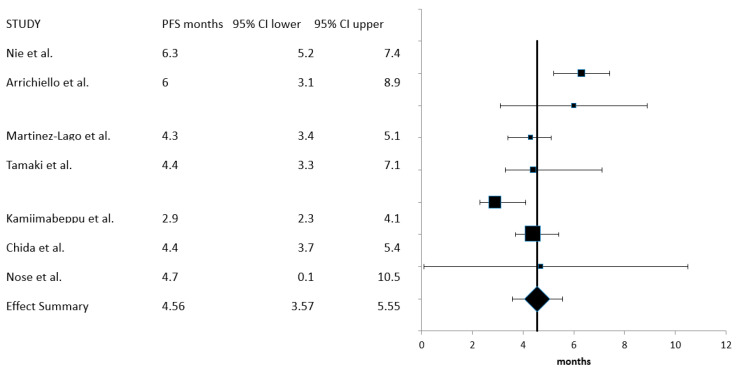
Meta-analysis of progression-free survival (PFS) [18,19,20,21,22,23,24].

Seven studies including 416 patients who had data on progression-free survival (PFS) available were entered in the PFS meta-analysis. Heterogeneity was high in this case (*I*^2^ = 74, Cochran’s Q = 23.3, x^2^ *p* = 0.0006), and thus, a random-effects model was employed. The summary PFS was 4.56 months (95% CI: 3.57–5.55 months) (Figure 4).

**Figure 5:**
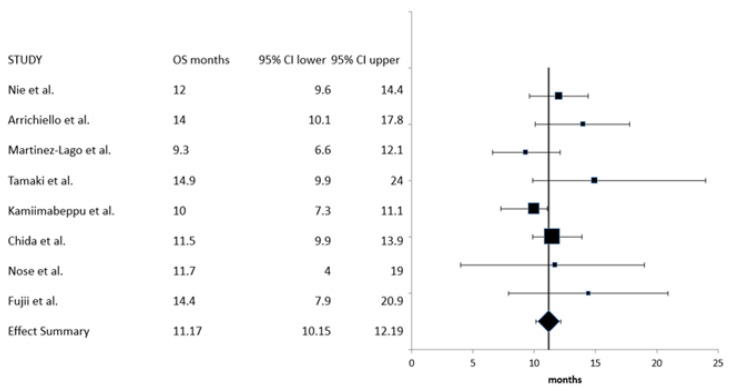
Meta-analysis of overall survival (OS) [18,19,20,21,22,23,24,25].

OS data were available from all eight studies, with a total of 437 patients. Study heterogeneity in this case was low (*I*^2^ = 11, Cochran’s Q = 7.9, x^2^ *p* = 0.34), and a fixed-effects model was used. The summary OS was 11.17 months (95% CI: 10.15–12.19 months) (Figure 5).

Regarding efficacy according to line of therapy, one of the two western studies included in the meta-analysis showed that patients with more than three lines of previous therapies had an inferior PFS of 3 months compared to PFS of 8 months in patients with three previous lines of therapy [19]. This difference was not statistically significant. The other western study and the three Asian studies, that included data on the number of previous lines of therapies, did not report efficacy results according to the number of previous lines of treatment received [18,20,21,22]. Similarly, no data on efficacy of treatment, according to whether patients had undergone surgery for the primary site, were reported.

Among the most frequent adverse effects of all grades of the combination were neutropenia, anemia, thrombocytopenia, nausea, fatigue, anorexia, diarrhea, proteinuria, and hypertension (Table 4). Grades 3 and 4 adverse effects occurring in more than 10% of patients were neutropenia (44.9%) and anemia (12.6%).

## 4. Discussion

Options for later-line treatment of metastatic colorectal cancer patients, after progression on fluoropyrimidines, oxaliplatin, and irinotecan chemotherapy, are few. The two approved treatments for this setting are the combination of trifluridine/tipiracil and the anti-angiogenic small-molecule multikinase inhibitor regorafenib [10,28]. These treatments produce only modest survival benefit. The OS of patients in the trifluridine/tipiracil arm of the RECOURSE randomized trial, for example, was 7.1 months, while, in the placebo arm of the trial, it was 5.3 months [13]. In the CORRECT trial that compared regorafenib with a placebo, the OS in the regorafenib arm was 6.4 months and, in the placebo arm, the OS was 5 months [29]. In another randomized study of regorafenib versus placebo, the CONCUR trial that included patients from Asia, the OS in the regorafenib arm was 8.8 months and, in the placebo arm, the OS was 6.3 months [30]. Based on the model of adding a targeted agent with a chemotherapy backbone in earlier lines of metastatic colorectal cancer treatment, the combination of trifluridine/tipiracil with an anti-angiogenic agent seemed a rational next step to improve on the efficacy of the chemotherapeutic [31]. Bevacizumab was chosen as the combination partner based on its record of additive efficacy and manageable adverse-effect profile. An initial single-arm phase 1/2 trial (the C-TASK FORCE trial) disclosed a PFS at 16 weeks of 42.9% (80% CI: 27.8–59%) and a manageable toxicity profile [32]. Another randomized phase 2 trial from Denmark suggested the superiority of trifluridine/tipiracil plus bevacizumab over trifluridine/tipiracil monotherapy in patients pretreated with a fluoropyrimidine, oxaliplatin, and irinotecan [33]. Median PFS was 4.6 months in the bevacizumab arm versus 2.6 months without bevacizumab. Results from the open-label, phase 3 SUNLIGHT trial showed improvement of the OS with trifluridine/tipiracil plus bevacizumab in comparison to oral trifluridine/tipiracil as monotherapy [14]. Median OS in the arm of bevacizumab was 10.8 months versus 7.5 months with trifluridine/tipiracil alone (*p* < 0.001).

The current systematic review and meta-analysis examines evidence for the efficacy and tolerability of the trifluridine/tipiracil plus bevacizumab combination regimen in later-line metastatic colorectal cancer from the reported experience in the oncology community outside the clinical trial setting. In contrast to previous meta-analyses, it explicitly considered only data from reports of retrospective series and excluded data from phase 2 and phase 3 trials, with the goal to confirm that trial data are similar with the “real world” experience [34,35]. In addition, several studies included in the current meta-analysis have been published after the previous meta-analyses. The current meta-analysis confirms that the trifluridine/tipiracil plus bevacizumab combination provides a limited number of objective responses in the later-line therapy setting of colorectal cancer. The RR of 2.71% in the meta-analysis is similar to the RR of 2.2% (1 of 46 patients) and 4% (1 of 25 patients) that was observed in the Danish and the C-TASK FORCE trials, respectively [32,33]. The RR was 6.3% in the trifluridine/tipiracil plus bevacizumab arm of the SUNLIGH trial [14]. However, also consistent with the two trials, the DCR was achieved in about three out of five patients. The DCR was 67%, 68%, and 76.6% in the Danish phase 2, the C-TASK FORCE, and the SUNLIGHT trials, respectively [14,32,33]. The OS observed in the meta-analysis of the series was 11.17 months, which is very similar to the OS of 10.8 months observed in the trifluridine/tipiracil plus bevacizumab arm of the SUNLIGHT trial [14]. The median OS with trifluridine/tipiracil alone in the SUNLIGHT trial was 7.5 months. This was similar to the median OS of 6.6 months observed in a meta-analysis of observational series of later-line metastatic colorectal cancer patients treated with trifluridine/tipiracil [36].

The performance of regorafenib, which is the other approved treatment of metastatic colorectal cancer in the same later-line setting, in real-world studies was similar to the regorafenib arms of corresponding phase 3 trials [11,29,30]. A meta-analysis of observational real-world studies of regorafenib disclosed a summary OS of 7.27 months, while the regorafenib arms of the randomized CORRECT and CONCUR trials showed median OSs of 6.4 months and 8.8 months, respectively [11]. Indirect comparisons of trifluridine/tipiracil monotherapy and regorafenib show similar efficacy of the two regimens [37]. Comparison of trifluridine/tipiracil with regorafenib in the real world showed equivalent overall survival [38].

Neutropenia was observed as the most common grades 3–4 adverse effect of trifluridine/tipiracil plus bevacizumab, with an incidence of 44.9%. Neutropenia was also the most frequent grades 3–4 adverse effect in the Danish phase 2 and the C-TASK FORCE trials, with incidences of 67% and 72%, respectively [32,33]. Grades 3–4 anemia, which was observed in 12.6% of patients in the meta-analysis, was present in 4% and 16% of patients in the Danish phase 2 and the C-TASK FORCE trials, respectively. The rate of grades 3–4 neutropenia and anemia in the in the trifluridine/tipiracil arm of the phase 2 trial was 38% and 17%, respectively [33]. The development of neutropenia of any grade in cycles 1 and 2 has been associated with the effectiveness of trifluridine/tipiracil [39]. It would be interesting to investigate whether this common adverse effect is also associated with the effectiveness of trifluridine/tipiracil plus bevacizumab. A biomarker of efficacy that has been proposed for the trifluridine/tipiracil plus bevacizumab combination is the pretreatment lymphocyte-to-monocyte ratio [40]. However, given that various neutrophil subsets and platelet ratios are prognostic of survival outcomes in metastatic colorectal cancer [41,42,43,44], it remains to be determined whether the lymphocyte-to-monocyte ratio is a genuine predictive biomarker of trifluridine/tipiracil plus bevacizumab treatment or a prognostic biomarker of the disease. Taken into consideration the generally poorer clinical status of pre-treated advanced colorectal cancer patients, identification of biomarkers of response to later-line treatments is an important goal to spare these patients the added toxicity of an ineffective therapy. 

There are limitations in the current meta-analysis of observational studies of trifluridine/tipiracil plus bevacizumab therapy. First, most of the studies available and included in the analysis are from Japan, and only two studies were performed outside Asia. As a result, whether findings are valid in other populations, such as Caucasians and Africans, is unknown. This is important, given the population differences in the prevalence of enzyme polymorphisms involved in trifluridine/tipiracil metabolism and efficacy [45]. However, the results of the two series from western countries included in the meta-analysis, as well as the results of trials that included mostly western populations, are reassuringly close to the overall effects of the meta-analysis. A second limitation of the current study is that most series were small, and the overall number of patients available for the meta-analysis was moderate. In addition, some of the studies provided no information for all outcomes of interest, further decreasing the total number of patients analyzed for the respective outcomes. Information on the efficacy of the trifluridine/tipiracil plus bevacizumab treatment in different subsets of patients, according to the number of previous lines of therapies received (e.g., three lines or more than three lines of previous systemic therapies) or according to the diagnosis of metastatic colorectal cancer as synchronous with the primary cancer or as metachronous disease, was not available. These different subsets may display variable benefit from the trifluridine/tipiracil plus bevacizumab treatment. The retrospective series included in the meta-analysis did not provide data regarding specific methods of radiologic follow-up of patients before and during the diagnosis of their metastatic disease nor did they comment on specific modes of local treatments for metastatic disease, such as previous metastasectomies. Improved radiologic methods for the timely diagnosis of colorectal cancer recurrence is important for better survival outcomes, and in fact, may provide improvements beyond the moderate benefits of combination chemotherapy in early or later-line treatments [46]. Similarly, improved methods of resection of oligometastatic disease can give the opportunity for longer survival with acceptable morbidity in a subset of patients [47].

In conclusion, data from the real world collected outside the clinical trial setting concur with the efficacy and tolerability of trifluridine/tipiracil plus bevacizumab as observed in clinical trials. Given these results and the efficacy results of randomized trials, the combination arises as the current most promising regimen in the third-line treatment of metastatic colorectal cancer.

## Figures and Tables

**Table 1:** The eight studies included in the meta-analysis of trifluridine/tipiracil plus bevacizumab in metastatic colorectal cancer patients. RR: response rate, DCR: disease control rate, NR: not reported.

Study [Reference]	Year of Publication	Country	Number of Patients	RR (%)	DCR (%)
Nie et al. [18]	2022	China	54	1.9	75.9
Arrichiello et al. [19]	2022	Italy	31	3.2	71.0
Martinez-Lago et al. [20]	2022	Spain	35 (31 evaluable for response)	3.2	51.6
Tamaki et al. [21]	2022	Japan	35	0	54.3
Kamiimabeppu et al. [22]	2021	Japan	94	0	44.7
Chida et al. [23]	2021	Japan	139	5.8	64.0
Nose et al. [24]	2020	Japan	32	NR	NR
Fujii et al. [25]	2020	Japan	21	0	76.2

**Table 2:** Patients’ characteristics and efficacy in patients from the retrospective studies. The third and fourth columns contain information on the total number of patients and the number of series the result depicted in the second column is based on. Percentages may not add up to 100 because of different denominators due to variable groupings or missing information in the pooled studies.

	Patients (%)	Total Patients with Data	Number of Series with Data
Age (median, range)	55–73 (26–83)	441	8
SEX			
Male	233 (52.8%)	441	8
Female	208 (47.2%)		
ECOG PS			
0–1	388 (92.4%)	420	7
>1	32 (7.6%)		
NUMBER OF PRIOR LINES OF CHEMO			
1–2	134 (53.8%)	249	5
>2	115 (46.2%)		
Range numbers	1–>4		
TYPES OF PRIOR CHEMOTHERAPY			
Fluoropyrimidine	354 (100%)	354	5
Irinotecan	338 (95.5%)	354	5
Oxaliplatin	350 (98.9%)	354	5
Anti-angiogenic	376 (95.7%)	393	7
Anti-EGFR	115 (32.5%)	354	5
LOCATION OF PRIMARY			
Colon (side not specified)	31 (57.4%)	54	1
Right-sided colon	98 (25.7%)	382	7
Left-sided colon	24 (27.6%)	87	3
Left-sided colon and rectum	227 (75.7%)	300	4
Rectum	41 (37.3%)	110	3
NUMBER OF ORGANS INVOLVED			
1	81 (25.2%)	321	5
2	72 (41.4%)	174	2
1–2	22 (62.9%)	35	1
≥2	118 (80.3%)	147	3
≥3	63 (30.1%)	209	3
SITES INVOLVED			
Lung	219 (64.6%)	339	5
Liver	213 (62.8%)	339	5
Peritoneum	96 (28.3%)	339	5
Lymph nodes	77 (45.6%)	169	3
SURGERY FOR PRIMARY			
Yes	187 (72.5%)	258	5
No	71 (27.5%)	258	5
EFFICACY			
Median OS (months) (95% CI)	11.17 (10.15–12.19)	437	8
Median PFS (months) (95% CI)	4.56 (3.57–5.55)	416	7
RR% (95% CI)	2.71 (1.11–4.32)	405	7
CBR% (95% CI)	59.63 (52.06–67.21)	405	7

**Table 3:** Molecular characteristics of cancers from the retrospective studies. The third and fourth columns provide information on the total number of patients and number of series that had data for each biomarker.

	Patients (%)	Total Patients with Data	Number of Series with Data
MSI			
pMMR/MSS	98 (81.7%)	120	3
dMMR/MSI	3 (2.5%)		
Unknown	19 (15.8%)		
KRAS MUTATION STATUS			
Wild-type	207 (46.9%)	441	8
Mutated	228 (51.7%)		
Unknown	6 (1.4%)		
NRAS MUTATION STATUS			
Wild-type	44 (81.5%)	54	1
Mutated	4 (7.4%)		
Unknown	6 (11.1%)		
BRAF MUTATION STATUS			
Wild-type	240 (92.7%)	259	4
Mutated	9 (3.5%)		
Unknown	10 (3.8%)		

**Table 4:** Toxicities of trifluridine/tipiracil plus bevacizumab in patients with metastatic colorectal cancer treated in later-line setting. The third and fifth columns present data regarding the total number of patients and number of series for all grades and grade 3/4 toxicities, respectively.

Toxicity	% All Grades	Total Patients with Data/Series with Data	% Grades 3 and 4	Total Patients with Data/Series with Data
Neutropenia	68%	281/6	44.9%	441/8
Anemia	43%	302/7	12.6%	420/7
Thrombocytopenia	28%	271/6	3.9%	389/6
Asthenia/fatigue	50.5%	208/6	3.7%	326/6
Anorexia	29.3%	215/4	0.3%	354/5
Diarrhea	28.4%	271/6	1.8%	389/6
Hypertension	15.6%	270/6	1.5%	388/6
Nausea/GI toxicity	53.9%	271/6	2.3%	389/6
Proteinuria	33.3%	267/6	3.9%	385/6
Hemorrhage	12.1%	124/3	0.8%	124/3

## Data Availability

All data related to this article are included in the report, and no additional data are available.
